# Characteristics of Ancient Shipwreck Wood from Huaguang Jiao No. 1 after Desalination

**DOI:** 10.3390/ma16020510

**Published:** 2023-01-05

**Authors:** Xinyou Liu, Lulu Zhu, Xinwei Tu, Changjun Zhang, Houyi Huang, Anca Maria Varodi

**Affiliations:** 1 Co-Innovation Center of Efficient Processing and Utilization of Forest Resources, Nanjing Forestry University, Str. Longpan No. 159, Nanjing 210037, China; 2 College of Furnishing and Industrial Design, Nanjing Forestry University, Str. Longpan No. 159, Nanjing 210037, China; 3 Faculty of Furniture Design and Wood Engineering, Transilvania University of Brașov, 500036 Brașov, Romania; 4 Advanced Analysis and Testing Center, Nanjing Forestry University, Str. Longpan No. 159, Nanjing 210037, China

**Keywords:** microscopy, waterlogged archaeological wood, SEM, XRD, FTIR, nanoindentation

## Abstract

Huaguangjiao I refers to the ancient Chinese wooden shipwreck of the South Song Dynasty (1127–1279 AD) discovered in the South China Sea in 1996. From 2008 to 2017, the archaeological waterlogged wood was desalted using deionized water combined with ultrasonic treatment, and desalted using EDTA-2Na, EDTAHO, and NaH_2_PO_4_·2H_2_O solutions. In this paper, the degree of degradation of the modified waterlogged archaeological wood and the moisture and content of the main components were determined. X-ray diffraction (XRD), Fourier transform infrared spectroscopy (FTIR), nanoindentation (NI), and scanning electron microscopy (SEM) were employed to investigate the state of wood degradation after desalination and desulfurization. The results showed that the water content of the wood was as high as 532~1149%, while the basic density was only 0.14~0.18 g/cm^3^, indicating that the wood had been seriously degraded. The holocellulose content was only 36–40%. Based on the XRD patterns, the degree of cellulose crystallinity in the modified wood was 14.08%. The elastic modulus and hardness of the ancient shipwreck wood after desalination and desulfurization were 1.28–4.31 and 0.10–0.28 GPa, respectively, according to nanoindentation. In addition, the FTIR spectra revealed that the biological deterioration of the modified wood caused cellulose and hemicellulose degradation, but no apparent lignin alteration occurred. The results could provide knowledge for appropriate dewatering, strengthening, and restoration strategies.

## 1. Introduction

Shipwreck Huaguangjiao No. 1 (Huaguang Reef No. 1) from the Southern Song Dynasty (AD1127—AD1279) was discovered in 1996 near the Huaguang Reef on the Xisha Islands (South China Sea; N 16°19′–16°22′, E 111°57′–112°06′) [1,2]. This ship was a merchant ship used for trade between China and Southeast Asian countries, and it hit the reef in bad weather [3]. Therefore, this is an essential source for studying China’s Maritime Silk Road during the Southern Song Dynasty. An archaeological excavation of the underwater site of the ancient ship “Huaguangjiao I” was conducted in 2008, yielding 511 wooden ship plates. 

Wooden archaeological artifacts excavated from the marine sources often contain problematic sulfur and salts, which can cause further degradation of ancient wood [4,5,6]. In wooden crafts, externally deposited metal sulfides and inherent organic sulfur are oxidized by atmospheric oxygen in the presence of water and/or iron ions, eventually forming sulfuric acid [7,8]. Most of wood plates excavated from Huanguangjiao I were covered by sediments. After removing these sediments, the ancient wood was detected to contain sulfur and iron compounds by XRD (X-ray diffraction) analysis, such as FeS_2_, Fe(OH)_2_, Na_2_SO_3_, K_2_SO_3_ and K_2_S, which are common in marine–archaeological wood [5,6]. Fe, K, Ca, Mg, Na, Al, Mn, Mo, S, and other cations were found in this wood analyzed by inductively coupled plasma atomic emission spectrometer (ICP-AES), among which the content of Fe was up to 65% [9]. Moreover, Cl^−^, NO_3_^−^, and SO_4_^2−^ anions were also found in the distilled water that had soaked in the wood. From 2008 to 2012, the archaeological waterlogged wood was desalted using deionized water impregnation combined with ultrasonic treatment until the Cl^−^ dropped below 0.05%; 98.9% of the Fe and 55.6% of the S were removed [8]. From 2013 to 2017, insoluble salt was desalted using EDTA-2Na, EDTAHO, and NaH_2_PO_4_·2H_2_O solutions [10]. Desalination and desulfurization are important steps in water-saturated archaeological wood, which can prevent the destruction of wood structure by acid substances produced by the oxidation of sulfur and iron compounds. Moreover, the study of the characteristics of ancient shipwrecks after desulfurization and desalting is a prerequisite for the restoration and conservation of these ancient shipwrecks. 

Ancient ship wood research has recently produced valuable findings [4,5,6,7]. It is necessary to assess the deterioration of archaeological wood before preservation. For a better understanding of the degraded wood, its state properties were assessed, such as wood density, cellulose microfibril angle [9,10,11], water absorption, holocellulose molar mass [12], decay rate [13,14], and cell-wall [15] ultrastructure. In addition, sulfur accumulation in underwater or underground environments accelerates the deterioration of wood, and desalination and desulfurization are often essential steps for restoring and protecting ancient ship wood [12,16]. The fragility of the degraded ancient ship wood cell wall indicates that different consolidation methods such as ethanol replacement, PEG replacement, freeze–vacuum drying, supercritical carbon dioxide, and other unique drying methods must be employed [16,17,18,19,20,21,22]. Many varieties of additives have been utilized to enhance the wood structures and avoid shrinkage or deformation, including PEG, trehalose, polyoctadecanol, epoxy resin, and other stable compounds [23,24,25]. To accurately assess the restoration and protection effects of ancient ship wood, scanning electron microscopy (SEM) [26,27], X-ray diffraction (XRD) [28], Fourier transform infrared spectroscopy (FTIR) [29], X-ray photoelectron spectroscopy (XPS) [30], thermogravimetric analysis (TGA) [31], and nanoindentation (NI) [32,33] are all commonly used. In short, research on the characteristics of ancient ship wood is the basis and premise for the research results described above, which provide knowledge for appropriate dewatering, strengthening, and restoration strategies.

This study analyzes the wood characteristics of the “Huaguangjiao I” ancient shipwreck after desalination. XRD, FTIR, NI, and SEM were employed to study the degradation state of the modified waterlogged archaeological wood’s degradation, and to determine the moisture and holocellulose content. The present study offers significant instructions for creating artificial ancient woods to restore and replace destroyed wood in the ancient wooden ships of China.

## 2. Materials and Methods

### 2.1. Materials

Five hundred eleven plates of waterlogged archaeological wood were extracted from the “Huaguangjiao I” ancient shipwreck. Based on their degree of degradation, three wood samples identified as *Pinus massoniana* with varying degrees of deterioration, obtained from the Hainan Museum, were selected for this study. These blocks are water-saturated, between 5.5 cm and 16.8 cm in length, soft like chocolate, and have brown and black color.

### 2.2. Determination of Moisture and Holocellulose Content

The maximum moisture content and basic density can be regarded as two scientific and comparatively more easily operated indicators of the degree of degradation of waterlogged wood [12,14,16,25]. The wood samples were stored in distilled water from 2017, after desalination, which is considered to have the highest moisture content. The maximum moisture of the three samples was determined using the GB/T1931–2009 method for determining wood moisture content [34] The GB/T1933–2009 method for determining wood density was used to determine the basic density [35]. 

In line with GB/T2677.6–94 Fibrous Raw Material—Determination of solvent extractives [36], was used to measure the concentration of the solvent extractives. In line with the fibrous raw material determination of acid-insoluble lignin, the concentration of acid-insoluble lignin was measured. Based on GB/T2677.10–94 Fibrous Raw Material—Determination of holocellulose [36], the concentration of holocellulose was measured.

### 2.3. X-ray Diffraction

After drying, the three degraded wood samples were ground to 80-mesh powder and pressed into three sample sheets (three wood samples for each case) at room temperature. The wood samples were investigated using in situ XRD with an X’ Pert Pro multipurpose diffractometer (PANalytical, Almelo, Netherlands) based on the equipment of the Rigaku Smart Lab 9 kWXRD system (Shimadzu Corporation, Kyoto, Japan); θ–2θ scanning was used to measure the effects of scattering intensity and angle. The angle range was 5–40°, with a 2°/min scanning speed. The spectrum provided was the average of three measurements for the new and used wood. The Segal method, the height of the (002) peak (I002, 2θ = 22.8°), and the minimum value between the (002) and (101) peaks (I_AM_, 2θ = 18°) were used to determine the crystallinity of cellulose by applying the following equation:

CR_x_ = (I_002_ − I_AM_)/I_002_ × 100% (1)

where CR_x_ (%) represents the degree of cellulose crystallinity, I_002_ represents both crystalline and amorphous materials, and I_AM_ indicates amorphous material.

### 2.4. Quasi-Static Nanoindentation Test

The sample used for the nanoindentation test must be parallel to the bottom and top (test surface), and the test surface must be smooth. The samples used in this experiment were first replaced with ethanol, dried at low temperature (35 °C) to MC = 12%, and then cut into 7 mm × 5 mm × 5 mm pieces; five specimens were prepared. All the samples were placed on the platform, and all angles were verified with a straight edge at 90°. Next, the samples were attached to a metal sample holder as pyramids. Finally, the apex was smoothed with a diamond knife using an ultramicrotome [37]. Nanoindentation tests were performed at ambient temperature and relative humidity (RH = 55%). An in situ nanomechanical test system with a diamond Berkovich tip (Hysitron TI980, Bruker, Ettlingen, Germany) was used. During the experiment, the loaded specimen was set to 5 s (maximum load = 400 nm), the holding time was 5 s, and the unloading time was 5 s. In addition, five indentations were tested for each specimen, totaling 25 measurements for each sample. Finally, the hardness (H) and elastic modulus (Er) were computed using the following formula described by Oliver and Pharr [38]:

(2) $$H=\frac{P_{max}}{A}$$

where H is the hardness, *P_max_* refers to the peak load determined at the maximum depth in the indentation cycle, and A is the projected contact area between the indenter and the sample.

In addition, the following presents the calculation of the reduced elastic modulus (Er) of the sample:

(3) $$E_{r}=\frac{\sqrt{\pi }}{2}\frac{S}{\sqrt{A}}\;$$

where *S* (stiffness) denotes the slope of the line of the unloading curve shown in the load–displacement plot, and *A* denotes the projected contact area. Furthermore, *S* was detected using a linear approximation of the high-load portion of the unloading curve (ranging from 90% to 70% of the load). 

### 2.5. Chemical Composition Analysis

The chemical composition changes in all three samples were investigated. Specimens from 3 samples were ground and passed through an 80-mesh sieve, and pressed into KBr pellets for FTIR investigation. The FTIR test equipment (Tensor 27, Bruker, Ettlingen, Germany) was chosen with a spectral resolution of 4 cm^−1^ within the range 4000 to 400 cm^−1^ for a total of 32 scans. Following the alignment of the light equipment, the background spectra were gathered before the measurement. FTIR spectra of each wood sample were recorded six times. Each spectrum was further processed for baseline correction and smoothing. An average spectrum of the six individually recorded ones was calculated. This average spectrum was further normalized (max–min normalization). These normalized average spectra of control and aged samples were further compared to highlight chemical changes due to the degradation. 

### 2.6. Morphological Characteristics

The current study used environmental SEM (Quanta 200, FEI Company, Eindhoven, Netherlands) to observe the wood specimen structures through electrical conductivity measurements to investigate potential changes from the physical perspectives of the ancient shipwreck wood samples. Wood samples were prepared by sputter gold coating (2 nm) based on gold–palladium SEM annular sputtering using a 2″ ID × 3″ OD × 0.1 mm Anatech (SC502–314; Quorum Technologies, Ltd., Watford, UK) target. Furthermore, it was found that the bombarding voltage adopted for the SEM could reach 20.0 kV.

## 3. Results

### 3.1. Determination of the Main Component Content

The maximum moisture is the absorbent capacity of the wood samples, which is related to their interior physical structure and is a direct way for evaluating the degree of deterioration [39]. These wood samples were stored in distilled water since 2017, when the desalination and desulfurization strategies were completed. Therefore, the wood samples had the highest moisture content. Table 1 lists the wood samples’ maximum moisture and basic density values. In general, the maximum moisture content of waterlogged wood can be divided into three grades [39]: severe degradation (maximum moisture concentration ≥400%), moderate degradation (maximum moisture concentration 185–400%), and mild degradation (maximum moisture concentration ≤185%). In addition, the basic density of the wood samples was between 0.143 and 0.176 g/cm^3^, which is only 30–40% of sound wood, indicating that “Huaguangjiao I” waterlogged wood exhibited severe degradation.

For waterlogged wood, to some extent, the content of the extract can reflect its environment and degree of degradation [39]. For example, Table 2 shows that the concentration of 1% NaOH extracted from waterlogged wood (12.75–14.26%) was significantly higher than that of sound wood [41]. In anoxic or nearly anoxic waterlogged conditions, biodeterioration of the wood is mainly bacterial, while more oxygenated environments additionally facilitate decay caused by soft-rot fungi [42]. Most of the components degraded by anaerobic bacteria are celluloses and hemicelluloses [42]. The low holocellulose content, 36.84–40.67%, indicates that celluloses and hemicelluloses were severely degraded. The lignin content in the wood samples was 59.86–63.89%, significantly higher than that of sound wood.

### 3.2. X-ray Diffraction

**Figure 1:**
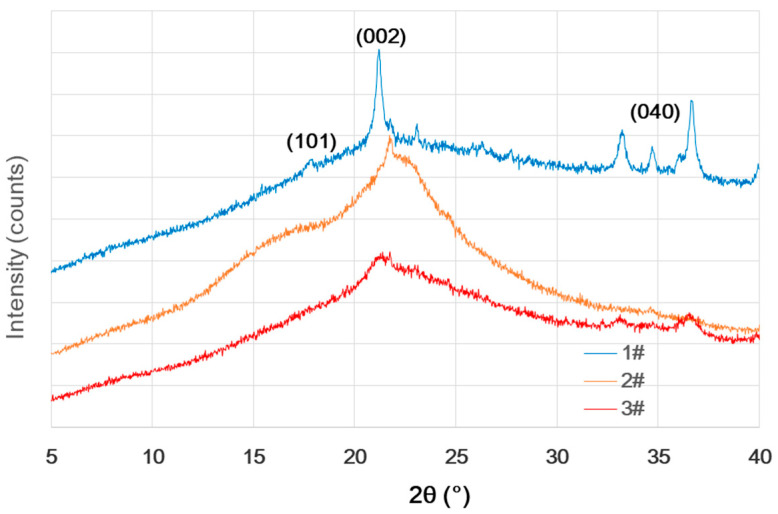
X-ray diffraction pattern of ancient shipwreck wood from Huaguang Jiao No. 1.

Cellulose is one of the three main components of wood, and its crystallinity reflects the structure of the cellulose microfilament, which is typically measured using an X-ray diffractometer. Figure 1 presents X-ray diffraction patterns of three ancient shipwreck wood samples from Huaguang Jiao I. The diffraction pattern of the 1# wood sample had three peaks at 2θ = 18°, 22.5°, and 35°, corresponding to the (101), (002), and (040) crystal planes, respectively. The other two patterns only have the maximum diffraction peak of (002) at 2θ = 22.8°, owing to severe degradation. By applying Equation (1), the calculated crystallinity index of cellulose for 1# wood sample reaches 14.08%, while that of sound wood is more than 40% [43]. In addition, the crystallinity of ancient wood decreases, which can be caused by the high partial loss of cellulose that results in reduced crystallinity [39].

### 3.3. Nanoindentation Test

**Figure 2:**
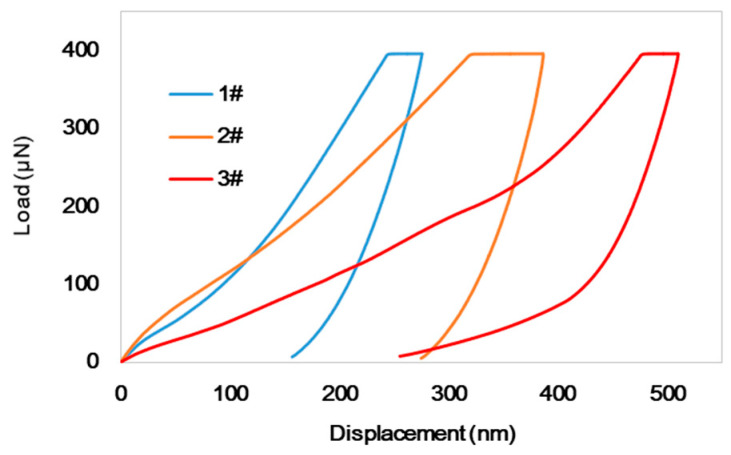
Typical NI load–displacement curves of the three degraded wood samples.

When the wood is susceptible, scarce, or minor, the nanoindentation test is the preferred mechanical test method. Nanoindentation can be used to monitor the load–displacement data of cell walls at the microscale, and the elastic modulus and hardness are the two standard parameters used to evaluate the mechanical properties of old wood [37]. Figure 2 presents the typical NI load–displacement curves of the three degraded wood samples. Compared with these curves, under the same load, the wood samples with a more severe deterioration state had more displacement, approximately twice that of the 1# wood sample, resulting in the curves for 2# and 3# shifted to the right. Table 3 presents the nanoindentation test results for the three degraded wood samples. The elastic modulus values are 4.31, 2.60, and 1.28 GPa, correspondingly; the hardness values are 0.28, 0.14, and 0.10 GPa, respectively. Compared with values of sound wood (elastic modulus = 7.57 GPa, hardness = 0.45 GPa) [43], the results illustrate that the degree of degradation significantly influenced the elastic modulus (*p* < 0.0001) and hardness (*p* < 0.0001).

### 3.4. Chemical Structure Analysis Using FTIR Spectroscopy

**Figure 3:**
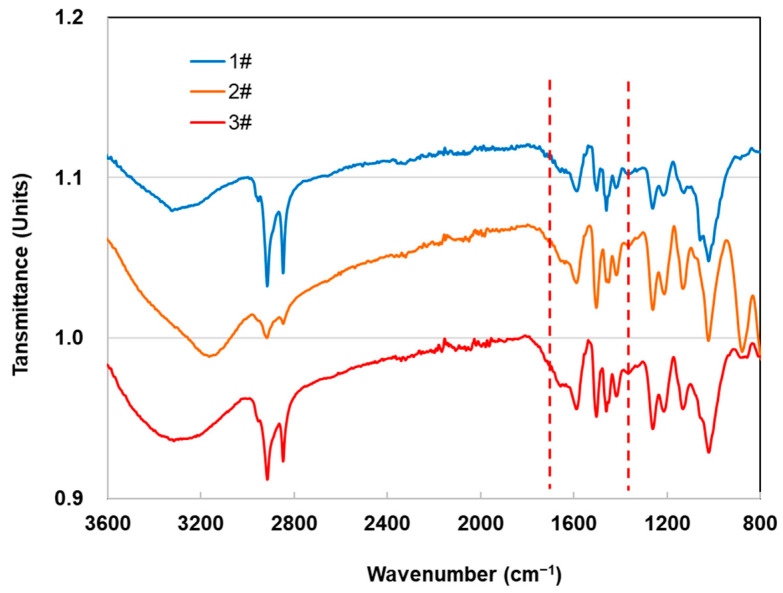
FTIR spectra of ancient shipwreck wood from Huaguang Jiao No. 1.

Figure 3 presents the FTIR spectra of ancient shipwreck wood’s three degrees of deterioration. All spectra showed strong bands between 3200 and 3400 cm^−1^, conforming to the stretching vibration of hydroxyl (–OH) [44], and C–H asymmetrical and symmetrical stretching at approximately 2880–2930 cm^−1^ [45]. These peaks also appeared to be identical to those of the sound wood. However, the spectra at approximately 1370 cm^−1^ due to CH deformation (symmetric) in carbohydrates and around 1730 cm^−1^ due to the C=O tensile vibration of xylan [46] had only tiny peaks, almost none, belonging to the stretching vibration of a carbon–oxygen double bond of a xylan acetyl group (CH_3_C=O), suggesting low hemicellulose and cellulose concentration in the ancient wood samples. In contrast, the peaks at 1602 cm^−1^, conforming to the C=C stretching of the aromatic ring (lignin), increase slightly. The peaks at 1510 cm^−1^ did not change significantly, indicating that the relative content of lignin in ancient wood could be increased [47,48], which means that lignin was only slightly degraded during the deterioration process, which is in agreement with previous studies [49,50,51].

### 3.5. Morphology

**Figure 4:**
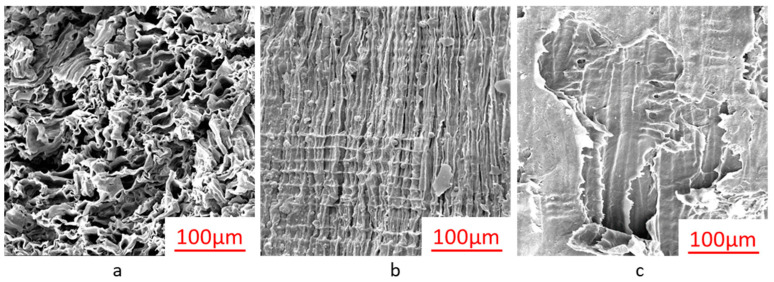
SEM micrographs of ancient shipwreck wood (sample 1#) in the transverse (**a**), radial (**b**), and tangential (**c**) sections.

Figure 4 shows SEM images of three different sections to assess the potential changes in the physical structure of ancient shipwreck wood after deterioration. Comparing the SEM images in our previous work, in the cross-section, the cell section appeared more mussy (Figure 4a) because of the deformation during drying and vacuuming caused by the fragile cell walls after cellulose and hemicellulose degradation. In the same situation, SEM micrographs in radial (Figure 4b) and tangential (Figure 4c) sections indicate severe degradation.

## 4. Conclusions

In conclusion, the investigated wood samples exhibited substantially greater maximum moisture content but a lower basic density than sound wood. An inverse linear relationship between the maximum moisture content and the basic density was observed due to the biodegradation of cellulose and hemicellulose in an underwater environment. The degree of crystallinity of cellulose was low at only 14.8%, which is much smaller than that of sound wood 41%. The components and FTIR spectral analysis showed that lignin was more stable, whereas the cellulose and hemicellulose in the wood samples were severely degraded. In addition, the wood samples lost their mechanical strength, as demonstrated by nanoindentation and SEM micrographs. The degradation state of the plates was basically the same for all 531 wooden plates excavated from the Huaguangjiao Reef No. 1 ancient shipwreck. The findings obtained in these studies could provide valuable information for the restoration and conservation of this ancient ship. 

## Figures and Tables

**Table 1:** Determination of maximum moisture and basic density.

Wood Samples	Maximum Moisture (%)	Basic Density (g/cm^3^)
1#	532.38 (45.28) ^1^	0.176 (0.006) ^1^
2#	721.19 (57.67) ^1^	0.155 (0.005) ^1^
3#	1149.38 (78.02) ^1^	0.143(0.006) ^1^
Sound wood	-	0.449–0.510 [40]

# indicates the serial number of the wood samples. ^1^ Data are presented as the average values of five samples (standard deviations in brackets).

**Table 2:** Chemical composition of ancient shipwreck wood from Huaguang Jiao No. 1.

Wood Samples	Alcohol–Benzene Extract (%)	1% NaOH Extract (%)	Acid Accumulator Insoluble Lignin (%)	Holocellulose (%)
1#	2.16	12.75	59.86	40.67
2#	2.08	13.44	60.77	39.26
3#	1.97	14.26	63.89	36.84

# indicates the serial number of the wood samples.

**Table 3:** Nanoindentation test results of the three different degraded wood samples.

Wood Samples	Elastic Modulus (GPa)	Hardness (GPa)
1#	4.31 (0.22) ^1^	0.28 (0.03) ^1^
2#	2.60 (0.17^1^	0.14 (0.01) ^1^
3#	1.28 (0.13) ^1^	0.10 (0.01)^1^

# indicates the serial number of the wood samples. ^1^ Average value of the 25 measurements and the standard deviations are shown in brackets.

## Data Availability

Not applicable.
